# Comment on “Analysis of Microarray-Identified Genes and MicroRNAs Associated with Idiopathic Pulmonary Fibrosis”

**DOI:** 10.1155/2018/4789035

**Published:** 2018-09-27

**Authors:** Chenyu Li, Shujuan Wang, Lin Che, Xianghua Wang, Yan Xu

**Affiliations:** Department of Nephrology, The Affiliated Hospital of Qingdao University, Qingdao, China

Recently, we read the article by Dr. Fan and colleagues, “Analysis of Microarray-Identified Genes and MicroRNAs Associated with Idiopathic Pulmonary Fibrosis” [1] which appeared in the 14 May 2017 issue of Mediators of Inflammation. Since the results of the article are very useful, we collected original data and used different bioinformatics methods to reanalyse the raw data; however, we get different results compared with those of the article, and we think that the author's methods in bioinformatics analysis are inappropriate.

We noticed that the author did not perform quality assessment for the microarray; therefore, we utilized Normalized Unscaled Standard Errors (NUSE) [2]. NUSE is a more sensitive measure than Relative Log Expression (RLE). If the analysts are skeptical about the quality of a chip in the RLE charts, that suspicion can easily be determined when using the NUSE diagram. The calculation of NUSE is actually very simple, it is the standard deviation of a chip relative to the standard deviation of the entire group. If the whole group of chips is reliable, their standard deviation will be very close and usually around 1. Therefore, if there is a problem with the quality of the chip, it will significantly deviate from 1, which will affect the NUSE values of the other chips in the opposite direction. Of course, there is a very extreme situation, that is, when most chips have quality problems but their standard deviation is relatively close, which also appears that the NUSE value of the qualified chips will be significantly deviated from 1.

We collected raw data from GSE32537 and GSE32538 from the GEO database and used the R tool to perform quality assessment of the microarray. Since the platform of GSE32537 is the Affymetrix Human Gene 1.0 ST Array, we used the oligo [3] package for quality assessment.  Figure 1 shows that GSM806284's NUSE is higher than 1.05, obviously, which means that GSM806284 is an unqualified sample and cannot be used for further analysis. For GSE32538 (Affymetrix Multispecies miRNA-1 Array), we used the AffyPLM [2] package for quality assessment.  Figure 2 shows that GSM806429's NUSE is around 1.05 which means that GSM806284 is an unqualified sample and also cannot be used for further analysis. In summary, quality assessment is a very important part of bioinformatics analysis. Performing the quality assessment and obtaining more accurate and convincing results of differentially expressed gene analysis is the basis for further analysis such as GO enrichment analysis and KEGG pathway analysis.

## Figures and Tables

**Figure 1:**
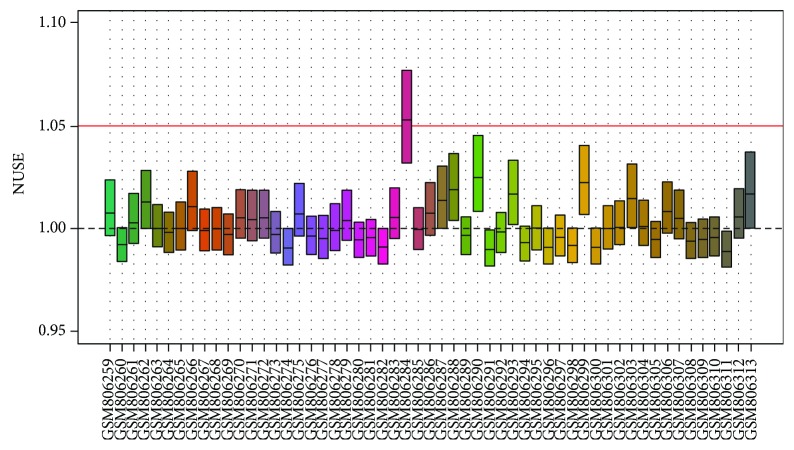
The NUSE plot of GSE32537.

**Figure 2:**
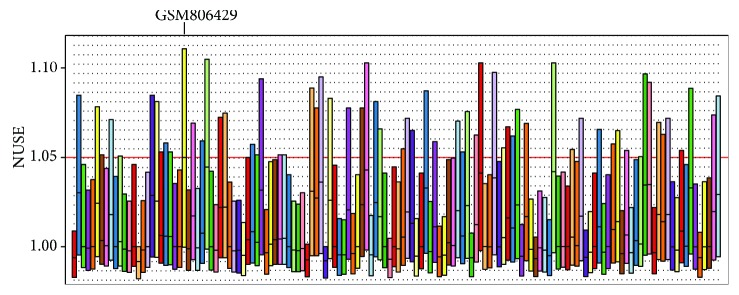
The NUSE plot of GSE32538.
